# Dopamine D_2_ gene expression interacts with environmental enrichment to impact lifespan and behavior

**DOI:** 10.18632/oncotarget.8088

**Published:** 2016-03-15

**Authors:** Panayotis K. Thanos, John Hamilton, Joseph R. O'Rourke, Anthony Napoli, Marcelo Febo, Nora D. Volkow, Kenneth Blum, Mark Gold

**Affiliations:** ^1^ Behavioral Neuropharmacology and Neuroimaging Laboratory on Addictions, Research Institute on Addictions, University at Buffalo, Buffalo, NY, USA; ^2^ Department of Psychology, Suffolk Community College, Riverhead, NY, USA; ^3^ Department of Psychiatry, University of Florida, Gainesville, FL, USA; ^4^ NIDA, Bethesda, MD, USA

**Keywords:** aging, D2, environmental enrichment, exercise, cognition, Gerotarget

## Abstract

Aging produces cellular, molecular, and behavioral changes affecting many areas of the brain. The dopamine (DA) system is known to be vulnerable to the effects of aging, which regulate behavioral functions such as locomotor activity, body weight, and reward and cognition. In particular, age-related DA D_2_ receptor (D2R) changes have been of particular interest given its relationship with addiction and other rewarding behavioral properties. Male and female wild-type (*Drd_2_* +/+), heterozygous (*Drd_2_* +/−) and knockout (*Drd_2_* −/−) mice were reared post-weaning in either an enriched environment (EE) or a deprived environment (DE). Over the course of their lifespan, body weight and locomotor activity was assessed. While an EE was generally found to be correlated with longer lifespan, these increases were only found in mice with normal or decreased expression of the D_2_ gene. *Drd_2_* +/+ EE mice lived nearly 16% longer than their DE counterparts. *Drd_2_* +/+ and *Drd_2_* +/− EE mice lived 22% and 21% longer than *Drd_2_* −/− EE mice, respectively. Moreover, both body weight and locomotor activity were moderated by environmental factors. In addition, EE mice show greater behavioral variability between genotypes compared to DE mice with respect to body weight and locomotor activity.

## INTRODUCTION

Dopamine (DA) is known to be implicated in a variety of functions including reward [1, 2] and physical mobility [3, 4]. The DA system has been known to be vulnerable to the effects of aging [5]. Human imaging studies have shown that the rate of D2R loss during aging occurs at approximately 10% per decade [6]. While it is apparent that D2R decreases in both human and rodent brains as a result of physiological deterioration following senescence, the functional consequences of this decline on behavior and lifespan are not fully understood [7-9].

The integrity of the DA system diminishes with age and contributes heavily to neurodegenerative diseases affecting motor output [10]. In particular, alterations in D2R have been associated as a primary mechanism for motor deficits [7, 11]. Several key areas of the brain are particularly vulnerable to the effects of aging: the substantia nigra (SNc), the ventral tegmental area (VTA), and the striatum. The SNc and VTA are the primary source for projecting DA neurons. DA neurons in the SNc project mainly to the dorsolateral striatum, forming the nigrostriatal pathway while DA neurons in the VTA project to the ventromedial striatum and the cortex, forming the mesolimbic and mesocortical pathways, respectively [12]. Decline in DA neuron populations within these areas, especially within the nigrostriatal pathway, has been shown to contribute to motor impairment and the progression of Parkinson's disease (PD) [13-16]. Additionally, decreases in SNc connectivity within the basal ganglia circuitry [17] as well as a decline in midbrain DA synthesis [16] have also been associated with PD. Moreover, a D2R deficiency has been known to lead to reduced spontaneous mobility and produce PD-like symptoms [18]. However, it should be noted that alterations in other DA receptor may also play an integral role in motor output. While traditional models understand DA-related locomotion through the cAMP pathway, modulated by the opposite functions of D1 and D_2_ receptors, it has been demonstrated that the PLC/IP3 pathway also contributes to locomotion exclusively through the activation of the D1 receptor; thus establishing the involvement of D1 receptor function in the discussion of PD [19].

D2R ability to modulate reward seeking behavior, motivation, and expectation of a reward, influences feeding behavior [20]. Alterations in the DA reward system can lead to abnormal eating behavior; the down regulation of D2R receptor signaling is thought to reduce sensitivity to reward, providing an incentive to overeat [21, 22]. This has been shown in human and rodent imaging studies, where obese subjects showed lower striatal D2R expression, which may pose as a risk factor for overeating [23-25]. Obese subjects showed a negative correlation between striatal D2R expression and body mass index [23]. Similar findings of reduced striatal D2R expression were also seen in rodent studies [25]. Reductions in D2R gene expression have also been associated with lower metabolism in the prefrontal cortex (PFC), which plays an important role in inhibitory control. The combination of reduced D2R signaling and reductions in prefrontal metabolism are thought to be powerful mediators with respect to the role of food intake and may contribute to obesity [24].

An EE is characterized by sensory, motor and social stimulation relative to standard housing conditions [26]. The incorporation of exercise is an important component of an EE and its benefits have been shown to be a powerful mediator of brain function and behaviour [27-29]. Furthermore, it has been shown that the benefits of an EE promote neurogenesis within the hippocampus [30, 31]. An EE also has the ability to modulate DA activity within certain areas of the brain; specifically, mice living in an EE show reduced DA release within the PFC [32, 33].

While it can be difficult to analyze which environmental components have influence over various domains of cognition, it is thought that the aerobic exercise component is a critical factor involved in the formation of memory [34]. Mustroph and colleagues has demonstrated in C57BL/6J mice that an EE, without the exercise component, failed to increase neurogenesis within the hippocampus and consequently improve cognition [34]. It is suggested that exercise may counteract some of the age-related deficits in metabolic support and neuronal dysfunction [35]. Moreover, research on the role of D2R in the aging process have revealed important polymorphic associations of the dopamine D2R gene and magnification of the aging process especially as it relates to memory and cognition [36, 37]. There is also research showing that polymorphisms of the D_2_ gene may influence the ability to be able to change ones goals as a function of environment. This flexible cognitive switching is an endophenotype of executive functioning and is highly heritable. Specifically, Markett and colleagues showed that carriers of the D_2_ A1+ variant who have a 30% decrease in lower striatal dopamine D_2_ receptor density compared to A1- carriers show a larger backward inhibition effect [38]. In line with previous results demonstrating increased behavioral flexibility in carriers of this genetic variant which favors cognitive flexibility may be important for cognitive manipulation, survival and lifespan.

To determine whether the D2R gene is involved in the mechanism of environmental enrichment, different housing conditions were examined in mice. This study hypothesized that the D_2_ gene, in the presence of an EE, significantly influenced lifespan, body weight, and locomotor activity.

## RESULTS

### Lifespan

Lifespan was measured in weeks and analyzed with a 2×3 Factorial ANOVA using sex, genotype and environment as the variable factors. Average lifespan for the entire population was 96.4 ± 1.43SE weeks, with the main effect of sex not being significant (*F*(1, 230) = .56; *p* > 0.05, η_p_^2^ = .002).

**Figure 1:**
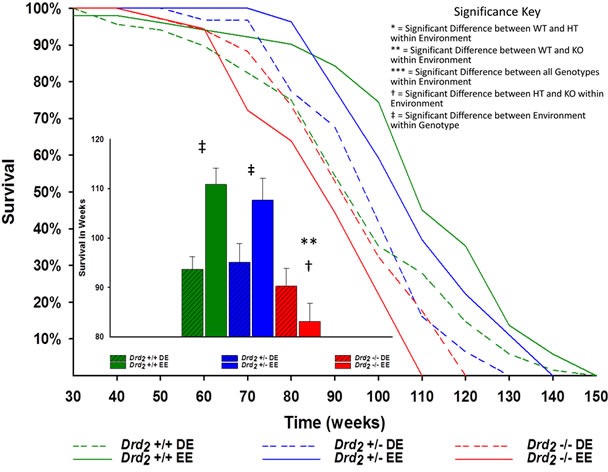
Main - Kaplan-Meier survival curves of mice for genotype and environment DE mice are dashed line and EE mice have solid lines. Drd2 +/+ are green, Drd2 +/− are blue and Drd2 −/− are red. Inset - Average lifespan ± SEM for each group. Drd2 +/+ EE mice lived more than 18% longer than their DE counterparts; ‡*p* < 0.0001. Drd2 −/− EE mice were found to have a 25% shorter lifespan compared to Drd2 +/+ EE; **p < 0.0001, and 23% shorter compared to Drd2 +/− EE mice; †*p* < 0.0001. The 13% increase in Drd2 +/− EE over Drd2 +/− DE was found to be significant; *p* = 0.05.

The main effect of genotype was significant (*F*(2, 230) = 12.3; *p* < .001, η_p_^2^ = 0.10; Figure 1), such that *Drd_2_* −/− had a 15.2% shorter lifespan than *Drd_2_* +/+ and 14.5% shorter lifespan than *Drd_2_* +/− (*p* < .05). Similarly, lifespan means for *Drd_2_* +/+ were significantly greater than *Drd_2_* +/− (*p* > .05). Environment also had a significant effect (*F*(1, 230) = 6.64; *p* > .05, η_p_^2^ = .03), such that EE mice lived 8.2% longer than DE mice.

The interaction of genotype x environment was also significant (*F*(2,230) = 7.00; *p* < .001, η_p_^2^ = .05). Between environments, *Drd_2_* +/+ mice significantly benefited from an EE, living more than 18% longer than their DE counterparts. The 13% increase in lifespan for *Drd_2_* +/− EE mice over their DE counterparts was found to also be significant (*p* = .05), whereas *Drd_2_* −/− lifespan was not significant between environments (*p* > .05) (see Figure 1). While the effects of genotype within a DE were not significant (*F*(2, 128) = .52 *p* > 0.05; η^2^ = 0.01), the effects of genotype in a EE were (*F*(2, 111) = 15.2; *p* < .001, η^2^ = .22), such that average lifespan for *Drd_2_* −/− EE mice was 25% shorter than *Drd_2_* +/+ EE and nearly 23% shorter than *Drd_2_* +/− EE mice (*p* < .0001 for both). Lifespans between *Drd_2_* +/+ and *Drd_2_* +/− EE mice were not significant (*p* > .05) (see Figure 1 and Supplement Table 1).

### Body weight

A 2 (sex) × 6 (time) mixed factorial ANOVA which weight was the dependent variable produced a significant main effect for sex (*F*(1, 273) = 216.42; *p* < 0.001, η_p_^2^ = 0.44). Overall, male mice weighed more than female mice, and interaction of sex and time revealed that males were heavier than females during every time point.

Subsequent analysis was employed with a repeated measure ANCOVA using sex as a covariate, and genotype, environment and time as variable factors. There was a significant main effect for time (*F*(5, 1365) = 158.2; *p* < .001, η_p_^2^ = 0.37) which was best explained by a quadratic effect (*F*_Quadratic_ (1,273) = 274.95, *p* < .001, η_p_^2^ = 0.50). For all groups, body weights rose during months 4 through 16, and then declined in months 20 and 24.

**Figure 2:**
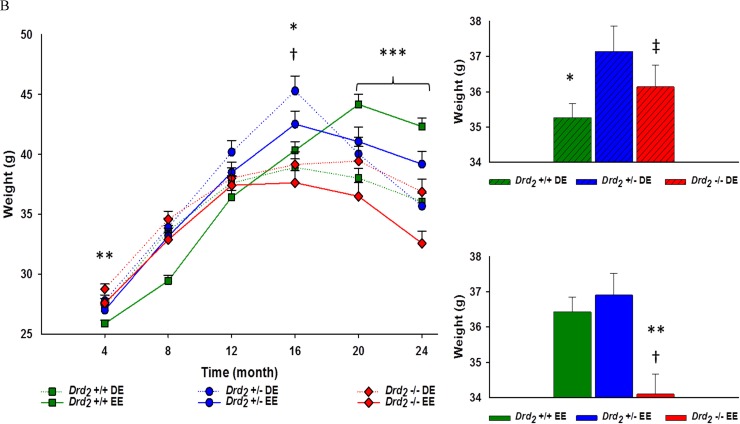
**A.** Main - Body weight for genotype, collapsing environment, at 4 month intervals for 24 months. Genotype × time interaction showed significantly different body weights at all time points; *Drd_*2*_ +/+ *vs*. *Drd*_*2*_ +/− *p* < 0.05, **Drd_*2*_ +/+ *vs*. *Drd*_*2*_ −/− *p* < 0.05, †*Drd*_*2*_ +/− *vs*. *Drd*_*2*_ −/− *p* < 0.05. Inset - Average body weight for each genotype ± SEM. *Drd*_*2*_ +/− had an overall 3% greater body weight than *Drd*_*2*_ +/+; *p < 0.05, and 5% greater body weight than *Drd*_*2*_ −/−; †*p* < 0.01. **B**. Left - Interaction of genotype × environment over time at 4 month intervals for 24 months. Significant differences between body weights were found at Month 4 and 16 DE mice, and Month 20 and 24 within EE mice; *Drd_*2*_ +/+ *vs*. *Drd*_*2*_ +/− *p* < 0.001, **Drd_*2*_ +/+ *vs*. *Drd*_*2*_ −/− *p* < 0.05*,* †*Drd*_*2*_ +/− *vs*. *Drd*_*2*_ −/− *p* < 0.05, ***Drd_*2*_ +/+ *vs*. *Drd*_*2*_ +/− *vs*. *Drd*_*2*_ −/− *p* < 0.001. Right - Average body weight for DE (Top) and EE (Bottom) for each genotype × environment. Within DE mice, *Drd*_*2*_ +/− had an overall 5% greater body weight than *Drd*_*2*_ +/+; *p < 0.05. Within EE mice, *Drd*_*2*_ −/− had an overall 6% lower body weight than **Drd_*2*_ +/+, and 8% lower than †*Drd*_*2*_ +/−; *p* < 0.001. Between environments, ‡*Drd*_*2*_ −/− within the DE had a 6% overall greater body weight than their EE counterparts. A 3% difference in body weight between DE and EE *Drd*_*2*_ +/+ were found to be approaching significance; *p* = 0.05.

The main effect of genotype was significant (*F*(2, 273) = 4.62; *p* < .05, η_p_^2^ = .03). *Drd_2_* +/− mice weighed an overall 3% more than *Drd_2_* +/+ (*p* < 0.05) and 5% heavier than *Drd_2_* −/− (*p* < .01) (see Figure 2A).

The interaction of genotype and time was significant (*F*(10, 1365) = 12.90; *p* = .001, η_p_^2^ = 0.09; Figure 2A). At 4 and 8 months of age, *Drd_2_* +/+ mice weighed less than *Drd_2_* +/− (*p* < .05) and *Drd_2_* −/− (*p* < 0.001). At 12 months of age, *Drd_2_* +/− had a greater body weight than *Drd_2_* +/+ (*p* < .01). *Drd_2_* +/− body weights peaked at 16 months of age and was more than 14% greater than both *Drd_2_* +/+ and *Drd_2_* −/− (*p* > 0.001 for both). At 20 and 24 months of age, *Drd_2_* −/− body weight dropped by 8% to 11% compared to *Drd_2_* +/+, and 6% to 7% compared to *Drd_2_* +/− (see Figure 2A).

The interaction of genotype × environment × time was significant (*F*(10, 1365) = 10.70; *p* < .001, η_p_^2^ = 0.07). However, the genotype × time interaction is greater (meaning more variation across genotypes) among the EE mice than the DE mice.

Within the DE, there was a significant effect for genotype for mice at 4 months of age, such that *Drd2* −/− weighed significantly more than *Drd_2_* +/+ (*p* < .01). All genotypes within the DE had equivalent body weights at 8 months of age, however, at 12 months, *Drd_2_* +/− had greater a greater body weight than *Drd_2_* +/+ (*p* < 05). At 16 months of age, *Drd_2_* +/− weighed significantly more than *Drd_2_* +/+ and *Drd_2_* −/− mice (*p* < 0.001 for both). However, at 20 and 24 months of age, *Drd_2_* +/− body weight dropped and all genotypes had equivalent weights (*p* > .05 for both time points)(see Figure 2B and Supplement Table 2). Within the EE, *Drd_2_* +/+ had lower body weights at 4 months and 8 months of age compared to *Drd_2_* +/− and *Drd_2_* −/− (*p* < 05). *Drd_2_* +/− body weight at 12 months of age was greater than *Drd_2_* +/+ (*p* < .05). At 16 months of age *Drd_2_* −/− body weight was less than both *Drd_2_* +/+ and *Drd_2_* +/− (*p* < .05). During 20 and 24 months of age, *Drd_2_* +/+ were heavier than *Drd_2_* +/− (*p* < .05), who were heavier than *Drd_2_* −/− (*p* < .01) (see Figure 2B and Supplement Table 2).

### Locomotor

A mixed design ANOVA using sex and time as variable factors showed both the main effect of sex (*F*(1, 278) = 0.024; *p* > .05, η^2^ < .0001), and the interaction of sex over time (*F*(5, 1390) = 0.501; *p* > .05, η^2^ < .002) was not significant. Therefore, the variable sex has been dropped, and the remaining ANOVAs used genotype, environmental enrichment and time as factors.

The main effect for time was significant (*F*(5, 1370) = 17.3; *p* < .001, η^2^ = .06), which was best explained by a quadratic effect (*F*_Quadratic_ (1, 274) = 38.3; *p* < .0001, η^2^ = .12). Activity was highest during 8 and 12 months of age, then declining through 16 to 24 months of age (see Supplement Table 3A).

**Figure 3:**
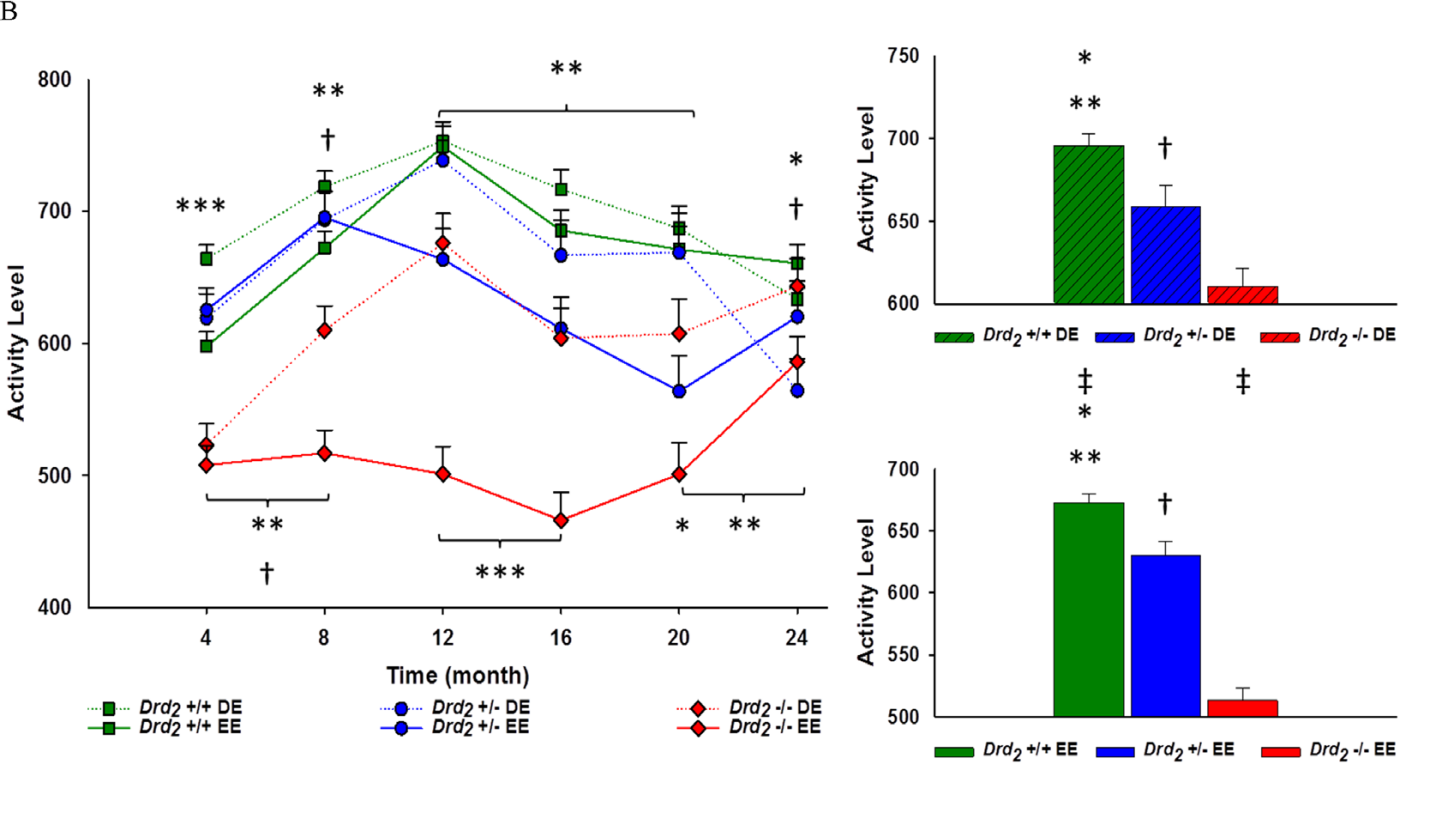
**A.** Main - Locomotor activity for genotype, collapsing environment, at 4 month intervals for 24 months. Genotype × time interaction showed hypoactivity among *Drd*_*2*_ −/− mice for all time points except Month 24, and *Drd*_*2*_ +/− compared to *Drd*_*2*_ +/+ following Month 12; *Drd_*2*_ +/+ *vs*. *Drd*_*2*_ +/− *p* < 0.01, **Drd_*2*_ +/+ *vs*. *Drd*_*2*_ −/− *p* < 0.0001, ***Drd_*2*_ +/+ *vs*. *Drd*_*2*_ +/− *vs*. *Drd*_*2*_ −/− *p* < 0.0001, †*Drd*_*2*_ +/− *vs*. *Drd*_*2*_ −/− *p* < 0.0001. Inset - Average locomotor activity for each genotype ± SEM. Overall locomotor activity for *Drd*_*2*_ +/+ were 6% greater than *Drd_*2*_ +/− and 18% greater than **Drd_*2*_ −/−. Overall activity for *Drd*_*2*_ +/− was also 13% greater than †*Drd*_*2*_ −/−; *p* < 0.0001 for all. **B**. Left - Interaction of genotype × environment over time at 4 month intervals for 24 months. Interaction of genotype × environment showed greater activity for *Drd*_*2*_ +/+ compared to *Drd*_*2*_ +/− DE mice at Month 4 and Month 24, and from Months 12 through 16 within the EE; *p < 0.05. Hypoactivity among *Drd*_*2*_ −/− lasted until Month 24 within the DE, and all time points within the EE compared to *Drd*_*2*_ +/+; **p < 0.05. *Drd*_*2*_ −/− had lower activity for both environments compared to *Drd*_*2*_ +/− and Month 4 and Month 8, and at Month 24 within the DE; ‡*p* < 0.05. *Drd*_*2*_ −/− EE remained hypoactive compared to *Drd*_*2*_ +/− EE up until Month 16; ‡*p* < 0.0001. Between environment, EE mice were hypoactive during Months 4 and 8 for *Drd*_*2*_ +/+, Month 12 and Month 16 for *Drd*_*2*_ +/−, and after Month 4 for *Drd*_*2*_ −/−; *p* < 0.005 (not shown). Right - Average locomotor activity for DE (Top) and EE (Bottom) for each genotype × environment. Within DE mice, overall locomotor activity for *Drd*_*2*_ +/+ was 5% greater than *Drd*_*2*_ +/−; *p < 0.05, and 12% greater than *Drd*_*2*_ −/−; **p < 0.0001. *Drd*_*2*_ +/− were 7% more active overall compared to *Drd*_*2*_ −/−; †*p* < 0.01. Results were similar for EE mice, *Drd*_*2*_ +/+ were 6% more active overall than *Drd*_*2*_ +/−; *p < 0.01, and 24% more active overall compared to *Drd*_*2*_ −/−; **p < 0.0001. *Drd*_*2*_ +/− were 19% more active overall compared to †*Drd*_*2*_ −/−; *p* < 0.0001. Between environments, *Drd*_*2*_ +/+ DE mice were 3% more active; ‡*p* < 0.05, and *Drd*_*2*_ −/− DE mice were 16% more active; ‡*p* < 0.0001, than their respective EE counterparts. Locomotor activity between *Drd*_*2*_ +/− environments was not significant; *p* > 0.05.

The main effect for genotype (*F*(2, 274) = 89.8; *p* < .001, η^2^ = .40), and the interaction of genotype × time (*F*(10, 1370) = 6.01; *p* < 0.001, η2 = .04) were significant. Overall, *Drd_2_* +/+ had the highest activity levels, and *Drd_2_* −/− had the lowest (*p* < .0001 between all genotypes) (see Figure 3A). From 4 to 20 months of age, activity levels for *Drd_2_* −/− were below *Drd_2_* +/+ and *Drd_2_* +/− (*p* < .05). From 12 to 24 months of age, *Drd_2_* +/+ were more active than *Drd_2_* +/−, (*p* < .05). At 24 months of age *Drd_2_* −/− activity was equivalent to both *Drd_2_* +/+ and *Drd_2_* +/− mice (*p* > .05) (see Figure 3A). The main effect of environment (*F*(1, 274) = 35.3; *p* < .001, η^2^ = 0.11) and the interaction of environment × time (*F*(5, 1370) = 5.73; *p* < .001, η2 = .02) were significant and assessed using independent sample *t*-tests. It was found that DE mice were significantly more active than EE mice for every month, except at 24 months of age (*p* > .05).

The interaction of genotype and environment was significant (*F*(2, 274) = 8.82; *p* < .001, η^2^ = 0.06). Between environments, *Drd_2_* +/+ and *Drd_2_* −/− DE mice were 3% and 16% more active than their EE counterparts. *Drd_2_* +/− activity was similar between environments (*p* > .05). A trend was seen where *Drd_2_* +/+ were more active than *Drd_2_* +/− (*p* < .05) and *Drd_2_* +/− were more active than *Drd_2_* −/− (*p* < .01) was seen within both environments (see Figure 3B). The interaction of genotype × environment × time was significant (*F*(10, 1370) = 3.50; *p* < .001, η^2^ = 0.03). Greater variation between genotypes existed in the EE (8.5%) compared to the DE (4.4%). Within the DE, *Drd_2_* +/+ had greater activity levels compared to *Drd_2_* +/− at 4 and 24 months of age (*p* < .05), while this was seen from 12 to 20 months of age within the EE (*p* < .01). *Drd_2_* −/− activity levels were lower than *Drd_2_* +/+ at every month, regardless of environment, except at 24 months of age where DE *Drd_2_* −/− had similar locomotor activity as DE *Drd_2_* +/+. *Drd_2_* +/− activity was greater than *Drd_2_* −/− in both environments at 4 and 8 months of age (*p* < .01). This continued within the EE for 12 and 16 months of age (*p* < .0001). (See Figure 3B and Supplement Table 3B).

Between environments and within genotype over time, *Drd_2_* +/+ DE activity levels were only greater than *Drd_2_* +/+ EE at 4 and 8 months of age (*p* < 0.01). Activity levels between *Drd_2_* +/− DE and EE were similar for all months, except at 12 and 20 months of age, where DE activity was greater than EE activity (*p* < .05). In contrast, *Drd_2_* −/− DE activity levels were greater than *Drd_2_* −/− EE after 4 months of age (*p* < .05) (see Figure 3B and Supplement Table 3B).

## DISCUSSION

### Lifespan/aging

Results supported the hypothesis and showed that the D_2_ gene along with environmental conditions combined to significantly influence lifespan, body weight, and locomotor activity. These data provide the first evidence of the role of D2R gene on lifespan in mammals (along with the mediating effects of environment) and are in agreement with very recent results by Landis et al in drosophila[39].

Mice with normal or reduced expression of the D_2_ gene and housed in an EE showed significant increases in lifespan. However, mice deficient in D_2_ failed to benefit from an EE. Similar increases in lifespan were also recently observed in D_4_ mice raised in an EE [40]. The D_2_ gene function appears to be a critical mediator linked to the behavior and lifespan effects associated with an EE. D_2_'s mediating role, however is environment-dependent and was not observed in mice raised in DE conditions.

**Figure 4:**
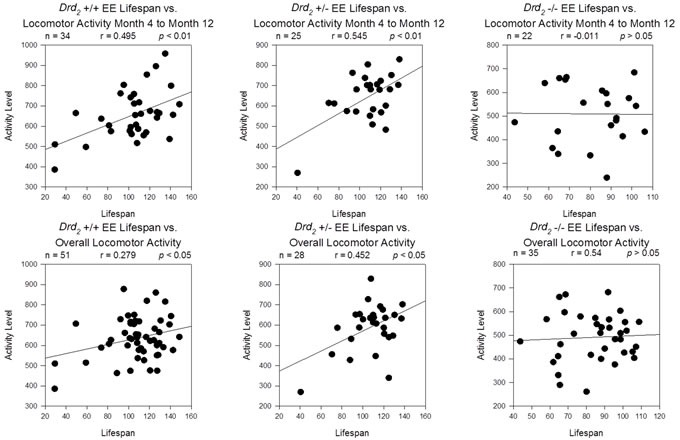
Top Row - Correlation of lifespan and average locomotor activity for Months 4 through 12 for *Drd*_2_ +/+ EE (Top Left), *Drd*_2_ +/− EE (Top Center) and *Drd*_2_ −/− EE mice (Top Right) A moderate and significant correlation was found for average locomotor activity in the first 8 months *vs*. lifespan for *Drd*_*2*_ +/+ EE (r = 0.495; *p* < 0.01) and *Drd*_*2*_ +/− EE (r = 0.545; *p* < 0.01), but not *Drd*_*2*_ −/− EE (*p* > 0.05). Bottom Row - Correlation of lifespan and overall average locomotor activity for the same groups produced similar results.

Mice exposed to EE, experienced social interaction, cognitive stimulation (enrichment toys in the cage), and exercise (access to a running wheel). Earlier studies have demonstrated the mediating effects of exercise on lifespan. For example, it has been shown that rats who exercised starting from 1.5 months of age displayed significant increases in lifespan compared to sedentary rats; male rats allowed to exercise lived 19.3% longer than male controls and female rats allowed to exercise lived 11.5% longer than female controls [41]. Interestingly, Samorajski and colleagues showed that mice who began exercise treatment at a later stage of development - both continuously or intermittently until death - did not show these increases in lifespan [42]. Lifespan benefits associated with exercise appeared to be dependent upon the age at which the exercise treatment was introduced. Locomotor activity within the first 8 months of age can be seen as a predictive factor in lifespan (See Figure 4). It is therefore possible that the lifespan differences between environment groups observed here could be attributed to the effects of early and continuous exercise.

More recent studies on exercise have displayed it's powerful health benefits, especially in relation to mitochondrial function which plays a critical role in many age-related neurodegenerative diseases [27, 43, 44]. The anti-aging and neuroprotective factors associated with exercise may be the key factor as to why *Drd*_2_ +/+ and *Drd*_2_ +/− EE mice showed increased lifespan in comparison to their DE cohorts. Interestingly, there is evidence for a powerful link between DA striatal function and longevity. This was made clear in an experiment showing the use of (−) deprenyl to counteract the age-related decline of nigrostriatal neurons [45]. Administration of either 0.25mg/kg of (−) deprenyl or saline solution was given three times a week to 2 year old rats; it was observed that (−) deprenyl-treated rats had an increased average lifespan of 197.98 ± 2.36 weeks compared to saline-treated rats with an average lifespan of 147.05 ± 0.56 weeks [45]. This is consistent with recent data in Drosophila showing that dopamine pathway genes including the D_2_ gene are candidate positive regulators of life span [39].

### Body weight

D2R Deficiency has been highlighted as one of the many components involved in Reward Deficiency Syndrome (RDS) which is characterized by polymorphic variations of certain genes which precede RDS, causing aberrant DA transmission in the mesocroticolimbic pathway, producing a dysfunction in brain reward [46-50]. It's possible that, given the strong connection between reward-seeking behavior and obesity [50, 51], an RDS-associated condition may underlie what is known as food addiction. Food addiction can be thought of as a manifestation of RDS, resulting in a self-compensatory mechanism for an imbalance of D2R expression within the striatum. The inability to adequately acquire the appropriate amount of reward from natural reinforcers may predispose an individual to obtain more than is necessary to reach satisfaction. This represents a non-homeostatic feeding mechanism where hedonic feeding acts as an overwhelming influence in achieving a sense of well-being and satisfaction (natural characteristics resulting from DA release). This is illustrative in the fact that *Drd_2_* +/− DE mice have significantly higher body weights than *Drd_2_* +/+ counterparts. The fact that this is not the case within an EE may indicate that there is an EE-associated protective factor which is attenuating the RDS impact on hedonic feeding in *Drd_2_* +/− mice.

Hormones are known to act on the CNS and regulate energy balance and eating behavior. These data include, among others, the effects of leptin and insulin receptor expression within midbrain DA neurons of the SNc and VTA [52]. These areas represent potential targets for insulin and leptin to influence the DA-related motivational circuitry associated with feeding. Kim and colleagues have shown that *Drd_2_* −/− mice have an increased sensitivity to leptin signaling, thus promoting reduced body weight and food intake [53]. This observation is seen in our data showing reduced weight in *Drd_2_* −/− compared to *Drd_2_* +/− and *Drd_2_* +/+ mice, but only within an EE. Additional evidence shows that leptin is able to modulate D2R expression [54]. There appears to be an inverted-U shaped curve between D2R expression and body weight within the DE. Since the D_2_ receptor is involved in motivational and goal-oriented behaviors, it is possible that lacking the D2R entirely may not provide mice with the incentive to overeat and become at risk for obesity. Also, leptin signaling in midbrain DA regions may reduce the nonhomeostatic properties of food which could result in a diminished motivation to eat [55]. Even more, acute infusion of leptin into the lateral ventricle of male wistar rats has been shown to reduce extracellular DA release in the NAc following food intake [56]. It's clear that there exists an intimate relationship between the D2R and leptin signaling; however this relationship remains far from completely understood especially with consideration to and interaction with environmental factors.

Opposite to the effects of leptin, the gastric hormone, ghrelin, alters homeostatic feeding behavior by stimulating hunger and food intake [57]. Ghrelin acts on the growth hormone secretagogue receptor (GHSR), which is known to be expressed in various locations including the hypothalamus, SNc, and VTA, and is implicated in the induction of feeding in both humans and rodents [58-60]. Recently, ghrelin has been shown to involve the DA system in the regulation of food intake and operant behavior. Evidence showing antagonism of D_1_, D_2_, and D_3_ receptors by pharmacological administration in Sprague-Dawley rats resulting in the prevention of hyperphagia from ghrelin illustrates how DA signaling is implication in food intake [61]. Also, administration of ghrelin into the VTA increased DA release in the NAc as well as locomotor activity [62]. Furthermore, it has been shown that ghrelin's influence in the mesolimbic pathway may be a required component for the integration of environmental cues and responding to reward expectation, thus promoting an orexigenic drive [63]. The regulation of sensing and responding to environmental food cues through the function of the VTA could therefore be influenced directly by metabolic hormones. Ghrelin appears to exert some of its appetite-stimulating influences by directly modulating the mesolimbic pathway, thus promoting increased motivation to eat [64]. While the interaction between ghrelin and the DA system is still not fully understood it is possible that lifelong differences in D2R expression may play a key role in regulating ghrelin's actions on feeding behavior.

Therefore these data suggest that environment can interact with D2R levels in producing effects on body weight; particularly within the EE. While *Drd_2_* +/− mice had overall a greater body weight than *Drd_2_* +/+ and *Drd_2_* −/− mice, this was primarily seen within an EE. Diminished D2R expression and it's correlation with higher body weight is consistent with obese humans data reporting a negative correlation with D2R expression [23]. A negative correlation has also been found between D2R expression and body weight in obese rats [65]. Since significant body weight differences among genotypes do not typically become apparent until the middle and late stages of lifespan, we could imply that alterations in metabolic signaling is associated with differences in D2R signaling and aging. The interaction between altered expression of the D2R and neuropeptides provides an explanation for lower body weight in *Drd_2_* −/− mice within an EE, but not for the *Drd_2_* −/− mice in a DE.

### Locomotor activity

In support of previous studies, D2R expression played a critical role on locomotor activity [18, 66-68]. However, the interaction between D2R and locomotor activity and environmental influence is poorly understood. Here EE mice showed 8.5 % variability between the genotypes across time while DE mice showed 4.4% variability between genotypes across time in terms of activity. Environment-related changes in locomotor activity seen in the current study are supported by previous studies which also show hyperactivity within DE mice [69-71]. Developmental adaptations to the environment are thought to involve exploratory mechanisms. D_1_ receptor activation and the subsequent release of acetylcholine in the PFC has been shown to play an important role in spontaneous motor output and exploratory behavior [72, 73]. There is evidence that the effects of prefrontal D_1_ stimulation and acetylcholine release are modified in rats reared in an EE [72]. An EE appears to reduce D_1_ function within the PFC and reduce acetylcholine release in response to stress, contributing to reductions in spontaneous motor activity, and that that rodents reared in enriched settings were more efficient explorers and habituated to novel environments faster [74]. The hyperactivity seen in the DE mice may be due to inefficient exploration of the testing field. Interestingly, *Drd_2_* −/− EE mice show a major reduction in locomotor activity compared to the *Drd_2_* −/− DE mice. This finding suggests that the proposed model for EE-induced hypoactivity appears to hold true for mice deficient in D2R. Thus, it is plausible to assume that alterations in D1 function within the PFC occur in the absence of D2R.

### Environment

Environmental conditions modify brain plasticity and induce changes on a cellular, molecular and behavioral level [75]. In order to understand the behavioral changes produced by an environment, it is necessary to look at the underlying neural mechanisms. Most interesting is the effect an environment has on the DA system. It has been established that novel and stressful stimuli within the environment can induce DA release within the PFC (possibly acting as a coping mechanism) *via* activation of VTA neurons [76-78]. It was further shown that stress-induced DA release in the PFC decreases with age [79, 80]. Additionally, it has been shown that the function of VTA DA neurons that project to the PFC are altered in rodents exposed to an EE; namely, stress-induced release of DA in the PFC was reduced in rats raised in an EE [81]. This is probably the result of a reduced perception of stress from the anxiolytic effects of an EE; in other words, an EE protects rodents so that they deal with future stressors more positively. Interestingly, previous reports have shown longevity to be correlated with stress reactivity to environmental factors between different strains of rats; namely, rats who were more behaviorally reactive to stress had a shorter lifespan. Since stressed rats showed earlier degeneration in dopaminergic pathways, it's thought that the shortened lifespan might be the result of a failure to adapt to environmental challenges leading to reduced compensatory adjustments in surviving neurons [82]. Although the current study did not directly compare reactivity to stress between environments, it is hypothesized that this model of environment-dependent behavioral stress reactivity may play a role in the longer lifespans of *Drd_2_* +/+ and *Drd_2_* +/− mice in an EE.

Lastly, it has been postulated that an EE may have protective effects against mitochondrial dysfunction. Exercise in particular has received much attention given its widespread health benefits and neuroprotective role in modulating reactive oxygen species (ROS). In accompaniment of aging, mitochondrial dysfunction is a critical mechanism known to occur which can leave an organism vulnerable to neurodegenerative diseases [83]. Exercise appears to be an ROS modulator *via* neurotrophins such as BDNF. Since BDNF is critical to the support and maintenance of neuronal populations, thus promoting healthy cognitive abilities, it is suggested that exercise can be protective in attenuating age-related cellular damage in diseases like Alzheimer's and Parkinson's disease [84]. The ability of sustained exercise to promote neuroplasticity combined with the anxiolytic effects of social interaction and environment enrichment setting, do promote the survival and healthy function of mice. The current findings support that D2R plays an important role and interacts with an enriched environment (that includes exercise) to promote lifespan.

### Limitations

The study of environmental enrichment is a complex phenomenon that can include multiple components such as type of enrichment stimuli (number of stimuli, novelty of stimuli), type of social interaction (play behavior, aggression, sexual behavior, social hierarchy), and exercise (forced *versus* voluntary). While many studies have characterized the individual contributions of each enrichment form, few, if any, have done so under the expressive differences of the D2R gene in rodents. Due to the fact that we are unable to distinguish between the individual component contributions of our designed enriched environment, we cannot determine which component is having a particular effect on the mice. It has yet to be established how these components operate in relation to D2R expression, thus further experimentation would be necessary in evaluating our enrichment components separately. Additionally, deleting a gene (as with our *Drd_2_* −/− mice) may result in compensatory changes in other neurochemical signaling pathways. These developmental adaptations may produce alternative behavioral characteristics which might be unaccounted for [66].

## CONCLUSIONS

The dichotomy over genes *versus* environment has provided a rigorous and long debate in deciphering individual differences in longevity. In truth, there exists a complex interaction among the two which contribute to such individual differences observed. In describing the significant contributions on longevity, a recent paper has demonstrated that the *Drd_4_* gene plays a critical role in modulating longevity within an EE [40]. The D4R, which is part of the same family of receptors as the D2R, appears to mediate lifespan similarly to D2R. While further research is needed into the specific environmental conditions, families of genetic polymorphisms and epigenetic mechanisms; these results provide the first evidence of D2R gene-environment interaction playing an important role in longevity and aging.

## MATERIALS AND METHODS

### Animals

Male and female D_2_ transgenic mice (breeding pairs were obtained from David Grandy of Oregon Health Sciences University, and bred as previously described [85, 86]), were split into 3 groups: mice that had either normal expression of the D_2_ receptor (*Drd_2_* +/+), half of the levels of expression of the D_2_ receptor (*Drd_2_* +/−), and mice that were deficient of the D_2_ receptor (*Drd_2_*−/−). These mice were placed in either an EE or a DE. The EE mice were group housed in a large cage (27 × 48 × 15 cm) with access to an igloo-shaped plastic container with a running wheel attachment and exploratory tunnels (Bio-Serv; Frenchtown, New Jersey, USA). The DE mice were single housed in a standard plastic cage (19 × 29 × 12 cm) without access to these enrichment objects. Mice from both environments were given bedding squares (Ancare; Bellmore, New York, USA). All mice were placed on an inverse 12 hour light cycle beginning at 0600 hours, with food and water given *ad libitum*. All experiments were approved by and conducted in compliance with Institutional Animal Care and Use Committee (IACUC).

### Lifespan

Daily health inspections were made to assess the health of the mice based on the use of a Body Condition Scoring (BCS) approach [87], where mice weights, spontaneous mobility, and the presence and treatments for any skin lesions associated with aging were present. Mice that were determined to be reaching their endpoint or in poor health were deeply anesthetized with 3% isofluorane followed by cervical dislocation and head decapitation; their brains were collected for future analysis.

### Body weight

Body weight measurements were taken in the middle of their dark cycle and conducted on a weekly basis. Body weight was binned into bi-monthly intervals starting at 16 weeks of age, and analyzed at 4 month intervals beginning at month 4 and ending on month 24.

### Locomotor activity

Locomotor activity was measured bi-monthly by placing the mice in an optical sensor acrylic arena (measuring 26.67 × 48.26 × 15.24 cm) for one hour. Data was collected for analysis by VitalView software (Minimitter Corporation, Oregon, USA) and was binned into 4-month intervals beginning at month 4 and ending on month 24.

### Analysis

Statistical analysis was performed using multiple repeated measures ANOVAs and ANCOVAs with SPSS software. Post hoc analyses were done using Fisher's LSD with significance level of 5% [α= 0.05].

### SUPPLEMENTARY MATERIAL TABLES


