# Supplementary material for: Dopamine D2 gene expression interacts with environmental enrichment to impact lifespan and behavior
Source: Oncotarget. 2016 Mar 15;7(15):19111–23. doi: 10.18632/oncotarget.8088 (PMC4991369; doi:10.18632/oncotarget.8088)
Supplement: Supplementary file 1 [file oncotarget-07-19111-s001.pdf]

Dopamine D_2 gene expression interacts with environmental enrichment to impact lifespan and behavior

Supplementary Material

Supplement Table 1: Raw data values for lifespan (in weeks) among genotype x environment sample size (*N*), average (*Mean*) ± standard error (*SE*), and the lower and upper bounds for 95% confidence interval (*95% CI*). Also see **Figure 1** Inset.

Genotype	*Environment*	*N*	*Mean*	*SE*	*95% CI*	
					Lower Bound	*Upper Bound*
Drd$_2$ +/+	DE	66	93.659	2.59	88.556	98.762
	EE	51	110.834	3.263	104.405	117.264
Drd$_2$ +/-	DE	31	95.112	3.753	87.717	102.507
	EE	26	107.659	4.419	98.952	116.366
Drd$_2$ -/-	DE	34	90.245	3.592	83.168	97.322
	EE	34	83.179	3.67	75.948	90.41

Supplement Table 2: Raw data values for body weight (in grams) among genotype x environment, at 4 month intervals for 24 months. Sample size (*N*), average (*M*) ± standard error (*SE*), and the lower and upper bounds for 95% confidence interval (*95% CI*). Also see **Figure 2.B** Left.

Genotype	*Time (month)*	*Deprived Environment*					*Enriched Environment*				
		N	*M*	*SE*	*95% CI*		*N*	*M*	*SE*	*95% CI*	
					Lower Bound	*Upper Bound*				*Lower Bound*	*Upper Bound*
Drd2 +/+	4	78	27.40	0.28	26.86	27.95	72	25.88	0.29	25.32	26.45
	8		33.62	0.45	32.75	34.50		29.45	0.46	28.54	30.36
	12		37.61	0.56	36.50	38.72		36.43	0.58	35.28	37.58
	16		38.91	0.71	37.50	40.31		40.33	0.74	38.88	41.79
	20		38.02	0.81	36.42	39.61		44.16	0.84	42.51	45.81
	24		36.05	0.70	34.68	37.42		42.32	0.72	40.90	43.73
Drd2 +/-	4	25	27.79	0.48	26.84	28.74	33	27.01	0.42	26.18	27.85
	8		33.90	0.78	32.37	35.43		33.16	0.68	31.83	34.50
	12		40.18	0.98	38.24	42.12		38.49	0.86	36.80	40.18
	16		45.29	1.24	42.84	47.74		42.52	1.09	40.38	44.66
	20		40.03	1.41	37.25	42.82		41.06	1.23	38.63	43.49
	24		35.68	1.21	33.29	38.07		39.18	1.06	37.10	41.27
Drd2 -/-	4	34	28.76	0.42	27.94	29.58	38	27.59	0.39	26.82	28.37
	8		34.59	0.67	33.28	35.91		32.88	0.63	31.64	34.12
	12		38.01	0.85	36.34	39.67		37.42	0.80	35.85	38.99
	16		39.15	1.07	37.05	41.26		37.63	1.01	35.64	39.62
	20		39.44	1.21	37.05	41.83		36.50	1.15	34.24	38.76
	24		36.88	1.04	34.82	38.93		32.58	0.99	30.64	34.52

Supplement Table 3A: Raw data values for average (*Mean*) ± standard error (*SE*) locomotor activity (beam breaks) at each 4 month time point. Locomotor activity was significantly different up until Month 16.

Month	*Mean**	*SE*	*95% CI*	
			Lower Bound	*Upper Bound*
4	589.6[a]	6.06	577.65	601.51
8	651.1[b]	7.07	637.23	665.07
12	680.3[c]	8.50	663.58	697.04
16	625.1[d]	8.59	608.15	641.97
20	616.6[d]	9.82	597.23	635.91
24	618.2[d]	7.84	602.58	633.46

*Means with different letters differ significantly (p <0.05)

Supplement Table 3B: Raw data values for locomotor activity among genotype x environment, at 4 month intervals for 24 months. Sample size (*N*), average (*Mean*) ± standard error (*SE*), and the lower and upper bounds for 95% confidence interval (*95% CI*). Also see **Figure 3.B** Left.

Genotype	*Time (month)*	*Deprived Environment*					*Enriched Environment*				
		N	*M*	*SE*	*95% CI*		*N*	*M*	*SE*	*95% CI*	
					Lower Bound	*Upper Bound*				*Lower Bound*	*Upper Bound*
Drd$_2$ +/+	4	78	664.20	10.50	643.60	684.90	73	597.80	10.90	576.50	619.20
	8		718.50	12.20	694.30	742.60		672.20	12.70	647.30	697.20
	12		753.20	14.70	724.30	782.20		749.20	15.20	719.20	779.10
	16		716.80	14.90	687.50	746.00		685.50	15.40	655.20	715.80
	20		687.10	17.00	653.60	720.60		671.20	17.60	636.60	705.80
	24		633.60	13.60	606.80	660.30		660.60	14.00	633.00	688.30
Drd$_2$ +/-	4	25	619.20	18.50	582.70	655.70	31	625.30	16.70	592.50	658.10
	8		693.80	21.60	651.20	736.40		695.40	19.40	657.20	733.70
	12		738.70	26.00	687.50	789.90		663.70	23.30	617.70	709.60
	16		666.80	26.30	615.10	718.50		611.20	23.60	564.70	657.70
	20		668.90	30.10	609.70	728.00		563.80	27.00	510.70	616.90
	24		564.30	24.00	517.00	611.50		620.30	21.50	577.80	662.70
Drd$_2$ -/-	4	34	523.10	15.90	491.80	554.40	39	507.90	14.80	478.70	537.10
	8		609.90	18.50	573.40	646.40		517.00	17.30	482.90	551.10
	12		676.00	22.30	632.10	719.80		501.10	20.80	460.20	542.10
	16		604.00	22.50	559.60	648.40		466.10	21.00	424.70	507.50
	20		607.40	25.80	556.70	658.20		501.00	24.10	453.70	548.40
	24		643.30	20.60	602.80	683.80		586.00	19.20	548.20	623.80
